# A Pilot Screening of Agro-Food Waste Products as Sources of Nutraceutical Formulations to Improve Simulated Postprandial Glycaemia and Insulinaemia in Healthy Subjects

**DOI:** 10.3390/nu12051292

**Published:** 2020-05-01

**Authors:** Gian Carlo Tenore, Domenico Caruso, Maria D’Avino, Giuseppe Buonomo, Giuseppe Caruso, Roberto Ciampaglia, Elisabetta Schiano, Maria Maisto, Giuseppe Annunziata, Ettore Novellino

**Affiliations:** 1 Department of Pharmacy, University of Naples Federico II, Via Domenico Montesano 49, 80131 Naples, Italy; giancarlo.tenore@unina.it (G.C.T.); roberto.ciampaglia@unina.it (R.C.); elisabetta.schiano@gmail.com (E.S.); maria.maisto@unina.it (M.M.); ettore.novellino@unina.it (E.N.); 2 Department of Internal Medicine, Hospital Cardarelli, Via Antonio Cardarelli, 80131 Naples, Italy; dr.domenicocaruso@gmail.com (D.C.); dott.mariadavino@gmail.com (M.D.); 3 Coop. Samnium Medica, Viale C. Colombo 18, 82037 Benevento, Italy; giuseppebuonomo@tin.it; 4 Department of Emergency, Hospital Cardarelli, Via Antonio Cardarelli, 80131 Naples, Italy; giuseppe.caruso@gmail.com

**Keywords:** glucose homeostasis, OGTT, abscisic acid, carotenoids, oleuropein, nutraceutical

## Abstract

The control of glucose homeostasis is the main goal for both the prevention and management of diabetes and pre-diabetes. Numerous drugs are available, despite their side effects. This is constantly leading people to be inclined to natural alternative treatments. Evidence indicates antioxidant-based nutraceuticals as an optimal tool for the glycaemic control. Currently, a great interest has been focused on the valorisation of agro-food by-products as sources of bioactive compounds including polyphenols. In this sense, we tested the efficacy of novel nutraceutical products based on polyphenolic extract from nectarines (NecP), tomato peels (TP), and olive leaves (EOL) on glycaemic and insulinemic responses. The three formulations contained, respectively, 0.007 mg abscisic acid (ABA)/g, 0.5 mg carotenoids/g, and 150 mg oleuropein/g. Twenty healthy subjects consumed a regular glucose solution (RG) or a treatment beverage (TB) obtained by mixing RG with the individual formulations (TB NecP, TB EOL, and TB TP), separately, and on different days. All three formulations significantly lowered the 30 min glucose plasma peak (*p* < 0.05 for all); similarly, NecP and TP also significantly lowered the 30 min insulin plasma peak (*p* < 0.05 for all). These results may lead to the hypothesis of a formulation of a multi-component nutraceutical with a synergistic efficacy for the glycaemic control.

## 1. Introduction

A wide range of natural substances of plant origin, specifically, polyphenols, carotenoids, and terpenoids [1,2,3,4,5] have been demonstrated to be active on glycaemia in humans. To this regard, agro-food waste products are increasingly attracting a great interest from the nutraceutical industry, since they represent still rich sources of bioactive compounds which can be conveniently recovered for the formulation of food supplements indicated for the control of glycaemia.

Fruit thinning is a widespread agronomical practice that involves removing excess unripe fruits, measuring 1–2 centimetres in diameter, to produce better-sized, ripe, and healthy fruits, albeit in smaller numbers. It is generally applied to a specific range of tree fruits, including apples, pears, plums, peaches, and nectarines, and consists of leaving a minimum of one fruit every 5–8 cm (plums and apricots) to a maximum of one fruit every 10–15 cm (apples and pears) and 20–25 cm (peaches and nectarines) on tree branches [6]. Since this practice may interest up to 40% of the entire tree fruit load, fruit thinning may lead to a massive agricultural waste product which is generally destined to fertilising or feeding. Interestingly, these waste fruits are supposed to be a significant source of abscisic acid (ABA). This phytohormone is majorly responsible for the regulation of plant growth and differentiation [7,8,9]. Specifically, the influence of ABA on fruit ripening has been well documented, although its mechanism of action is still unclear. Studies have revealed that there is a progressive accumulation of ABA during fruit ripening, reaching its maximum concentration at a specific stage after full bloom and then decreasing to its minimum level at the fruit fully ripe/harvest stage [10,11,12,13,14]. Abscisic acid is known as a suppressive of plant growth regulator, inducing expression of cell cycle inhibitors effective on DNA [15] and protein synthesis [16] and thereby arresting cell divisions [17] and blocking cell cycle progression at the initial stages [18]. Probably, this effect would be highly requested by the fruit at an immature stage when cell cycle progression can be disturbed by several environmental factors such as oxidative stress [18]. Later, the production of increasing levels of protective compounds, such as antioxidants, would make possible the completion of fruit development, so that the action of ABA is no longer required [18]. The plant hormone ABA is also produced by pancreatic β-cells [19], adipocytes and myoblasts [1] in response to glucose and active in humans. The most recent studies concerning the mechanism of interaction of ABA with the receptor lanthionine synthetase C-like 2 (LANCL2) in glycaemic control and their influence on insulin and glucagon-like peptide 1 (GLP-1) release highlight a leading role of ABA in the physiological regulation of plasma glucose levels in humans [20]. Plasma levels of ABA increases in healthy individuals administered with a concentrated glucose solution thus indicating the capacity of incretins to stimulate the release of ABA, similar to their effect on insulin secretion [1]. The mechanism at the base of the hypoglycaemic action of supplemented ABA in vivo at low doses (few micrograms compared to hundreds milligrams/kg body weight) would depend on its upregulating effects of glucose transport receptors, rather than its stimulatory capacity of insulin release [10]. Therefore, the administration of ABA at low doses may be suggested as a useful tool for the improvement of glucose tolerance in diabetic patients with deficiency of or resistance to insulin. To this regard, the fruit thinning waste product may be considered as a more convenient agro-food matrix for nutraceutical applications. Moreover, these formulations, as a source of ABA, would favour the intake of this human endogenous hormone, contributing to its plasma levels and, thus, to its physiological capacity to modulate glycaemia.

Olive leaves are a massive agricultural by-product of the harvesting or processing technology of olive fruits. They contain high amounts of polyphenols of which the many beneficial properties to human health of olive leaf extracts, used in traditional medicine, have always been ascribed. Specifically, oleuropein, one of the most abundant constituents of olive leaf extract, has been referred to as able to counteract oxidative stress correlated to plasma glucose levels thus indicating its ability to improve postprandial glycaemia [21,22]. Oleuropein seems to improve postprandial glycaemia, by counteracting Nox2-mediated oxidative stress, recognized as being majorly responsible for the cellular production of reactive oxygen species (ROS) [2]. Reactive oxygen species are indicated as being able to activate dipeptidyl peptidase-4 (DPP-4) which promptly disables incretin activity, thus unbalancing insulin secretion [23,24]. However, a specific role of oleuropein on insulinaemia has recently been elucidated. Carnevale et al. [25] demonstrated that 20 mg of pure oleuropein was able to lower postprandial glycaemia in healthy subjects by enhancing DPP-4 activity, plasma GLP-1 and insulin levels.

Tomato industrial processing originates a huge amount (up to 3% by fresh fruit weight) of industrial by-product (tomato pomace), consisting mainly of peels, seeds and pulp. Tomato pomace has no commercial value and is currently disposed in landfills and only partially recovered by drying or composting for the production of animal feed. Nevertheless, the abundance of several bioactive compounds, especially carotenoids (mainly, lycopene), up to five times the concentration in the pulp, suggests the possibility of employing the tomato pomace as a cheap and sustainable source, for the extraction of these valuable natural substances. Recent studies including clinical evidence of the bioactive properties of carotenoids have shown that these compounds may play a significant role in the treatment of diabetes by improving insulin resistance which has been indicated as a major risk factor for the development of type 2 diabetes mellitus (T2DM) [26]. In vivo experimental data have demonstrated the capacity of lycopene to improve glycaemia as well as other metabolic disorders in mice given a high-fat diet after its twelve-week oral supplementation, either as a pure compound or as tomato powder, at the same dosage [27]. Interestingly, previous human trials have indicated a linear correlation between plasma lycopene and β-carotene concentration and insulin sensitivity in healthy volunteers [28,29]. Specific carotenoids would act as peroxisome proliferator-activated receptor gamma (PPARγ) agonists, through a similar mechanism to thiazolidinediones, a class of oral antidiabetic drugs, which are adopted in clinical therapy [30]. It has also been observed that carotenoid intake has an inverse relation with glycosylated haemoglobin (HbA1c) levels [31]. To date, the molecular mechanisms at the base of the effects of carotenoids on glycaemia and insulinaemia remain unclear. Nevertheless, there is general agreement that the beneficial effects of carotenoids in diabetes cannot simply be associated with their antioxidant properties.

The first aim of the present work was to formulate pilot nutraceutical products based on a water extract of unripe fruits derived from fruit thinning (fruit thinning waste products, FTWPs); ethanol extract from olive leaves (EOL); and dried tomato peel powder (TP). Their exact contents of ABA, oleuropein, and carotenoids, respectively, were detected. Then, each formulation was tested on healthy human subjects in order to evaluate its effects on glycaemic and insulinemic responses to a standard glucose drink.

## 2. Materials and Methods

### 2.1. Reagents and Standards

All chemicals and reagents used were either analytical-reagent or high-performance liquid chromatography (HPLC) grade. The water was treated in a Milli-Q water purification system (Millipore, Bedford, MA) before use. (±)-2-Cis-4-trans-abscisic acid (ABA), cartridges Discovery SPE DSC-MCAX (bed wt, 300 mg; volume, 6 mL; Supelco Analytical, Bellefonte PA, USA), Supelclean SPE LC-NH_2_ (bed wt, 300 mg; volume, 6 mL; Supelco Analytical), acetone, ammonium acetate, hexanes, methanol (MeOH), methyl tert-butyl ether (MtBE), β-Carotene (≥95%), and lycopene (≥90%), oleuropein (≥98%) were all purchased from Sigma–Aldrich (Milano, Italy). Glucose syrup 75 g/150 mL was provided by Sclavo Diagnostics International S.r.l. (Sovicille, Siena, Italy).

### 2.2. Sample Collection and Sample Preparation for HPLC Analyses

The FTWPs (apples, pears, plums, peaches, and nectarines) were collected in June 2018 at the orchards of “Giaccio Frutta” society (Vitulazio, Caserta, Italy, 41°10′ N–14°13′ E), at 20–25 days after full bloom, coinciding with the fruit thinning stage. Tomato peels (cultivar San Marzano) were provided in September 2018 by La Torrente S.r.l. (Angri, Salerno, Italy, 40°43′ N–14°33′ E). Olive leaves (cultivar Ravece) were provided in September 2018 by Agriturismo Petrilli (Flumeri, Avellino, Italy, 41°4′ N–15°9′ E). All the procedures performed to obtain both the extract and the samples for the HPLC analyses are described in detail in the Supplementary Materials.

### 2.3. HPLC-DAD Analyses of Samples

The chromatographic apparatus consisted of a Jasco Extrema LC-4000 system (Jasco Inc., Easton, MD) provided with the following modular components: a vacuum degassing unit, a quaternary pump, an autoinjector, a column oven, and a diode array detector photodiode array detector (DAD). The ABA was determined according the method described by Bosco et al. [32] with slight modifications [32]. Carotenoids were analysed as previously described by Cooperstone et al. [33]. Determination of oleuropein in olive leave extracts was carried out as previously described by Cooperstone and colleagues (2016) [34]. Information about the HPLC-DAD system used and method are detailed in the Supplementary Materials.

### 2.4. Nutraceutical Product Preparation

Large-scale production of the nutraceutical products from agri-food waste matrixes was accomplished by MBMed Company (Turin, Italy).

Among all of the FTWPs, as the objects of this study, nectarines were found to be the richest in ABA content (Table 1). Thus, they were chosen as the ideal candidate to be used for the industrial transformation. Nectarines were extracted with water at 50 °C. After centrifugation, the extract underwent a spray-drying process with maltodextrins as support, obtaining a fine powder, containing an extract:maltodextrins ratio 1:1 (w/w) (nectarine dry extract powder, NecP).

Tomato peels were dried at 42 °C in industrial ovens to obtain a fine powder (dried tomato peel powder, TP).

Olive leaves were extracted with pure ethanol at room temperature. After centrifugation, the extract underwent a spray-drying process with maltodextrins as support, obtaining a fine powder, containing an extract:maltodextrins ratio 1:1 (w/w) (ethanol extract from olive leaves, EOL).

### 2.5. HPLC-DAD Analyses of Nutraceutical Products

Chromatographic analyses for the determination of ABA, carotenoids, and oleuropein levels in the nutraceutical products based on NecP, TP, and EOL, respectively, were conducted as reported in Section 2.3. The results are shown in Table 2.

### 2.6. Study Population and Protocol

This was a randomised, single centre, double-blind trial. The study was conducted on 18–70 years, normal-weight, and normal-glycaemic subjects recruited by the Samnium Medical Cooperative (Benevento) in January 2019. Participants completed six test sessions, each on a different day with consecutive sessions, separated by at least 1 week. Each participant tested the oral glucose solution on sessions 1, 3, and 5, and one of the three treatment beverages during each of the remaining sessions in a random, counterbalanced order. Participants consumed the reference glucose solution on three separate occasions and each test beverage on one occasion only. Subjects were informed not to drink alcohol or perform hard physical activity 48 h prior to blood sampling. Participants maintained their usual dietary and lifestyle patterns throughout the study. The reference glucose solutions and the treatment beverages all contained 75 g glucose. The three treatment beverages (TBs) were prepared by mixing the glucose solutions with the following samples: 2 g of NecP (14 µg ABA) → TB NecP; 2 g TP (1.0 mg total carotenoids) → TB TP; 400 mg EOL (60.0 mg oleuropein) → TB EOL. Both TB and reference glucose solutions were served into dark jars, in order to blind subjects and researchers of the study to the different colours of the solutions mixed with the nutraceutical products. The nutraceutical products required to prepare each treatment beverage were added to the glucose solutions immediately before being served to the subjects. The study was conducted in accordance with the 1964 Helsinki Declaration (revised in 2000) and approved by the Scientific Ethics Committee of AO Rummo Hospital (Benevento, Italy) with protocol no. 28 of 15 May 2017. Additional information concerning the study protocol, including study procedures and statistical analyses, are detailed in the Supplementary Materials.

## 3. Results

### 3.1. Enrolment

A total of 20 healthy subjects (11 women and 9 men) with a mean age of 45.1 ± 15.8 years and an average BMI of 23.3 ± 3.4 kg/m^2^ were assigned to the study (Table 3). The group was well balanced for demographics and clinical factors. No subject prematurely terminated study participation. All participants performed the six test sessions (dropout rate: 0%). The mean within-individual coefficient of variation for the glycaemic responses to the three repeated glucose solutions was 11% which was within the accepted level of ≤30% (ISO 26642:2010).

### 3.2. Tolerability of Treatment Beverages

The TB were palatable and well tolerated. No adverse events were reported.

### 3.3. Glycaemia and Insulinaemia Responses to Reference Glucose Solution and Treatment Beverages

**Figure 1:**
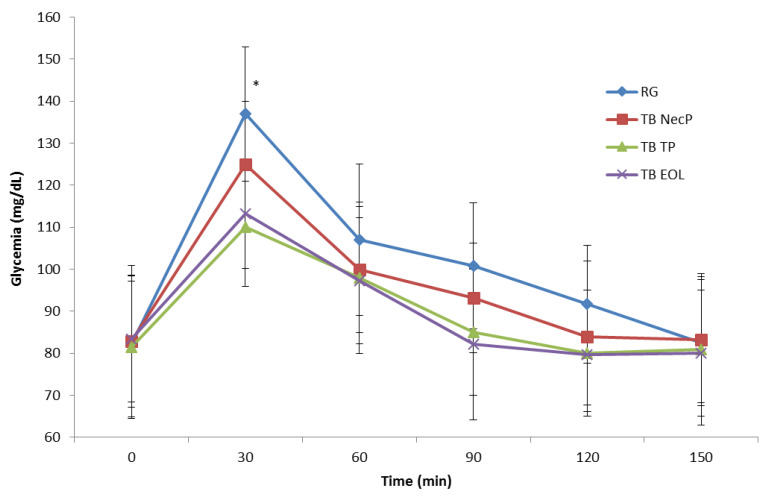
Change in postprandial plasma glucose concentration in healthy adults for the three treatment beverages. TB NecP (2 g of NecP, equivalent to 14 µg ABA); TB EOL (400 mg EOL, equivalent to 60.0 mg oleuropein); TB TP (2 g TP, equivalent to 1.0 mg total carotenoids). Data are mean ± SD. * Indicates a significant difference between peak 30 min glucose concentration for each treatment test compared to the reference test (*p* < 0.05). Abbreviations: RG, regular glucose solution; NecP, nectarine dry extract powder; TP, dried tomato peel powder; EOL, ethanol extract from olive leaves.

All of the three TB revealed lower peak plasma glucose concentrations at 30 min compared to the reference glucose solution (TB NecP, *p* = 0.02; TB TP, *p* = 0.02; TB EOL, *p* = 0.02) (Figure 1). Particularly, TB TP, and TB EOL demonstrated higher effects respect to TB NecP (*p* = 0.02 and *p* = 0.02, respectively), showing no significant difference among each other (*p* = 0.48).

**Figure 2:**
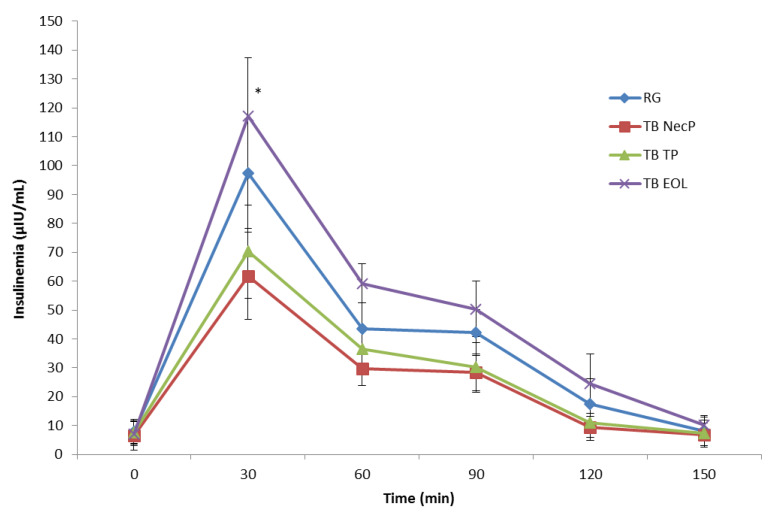
Change in postprandial plasma insulin concentration in healthy adults for the three treatment beverages. TB NecP (2 g of NecP, equivalent to 14 µg ABA); TB EOL (400 mg EOL, equivalent to 60.0 mg oleuropein); TB TP (2 g TP, equivalent to 1.0 mg total carotenoids). Data are mean ± SD. * Indicates a significant difference between peak 30 min insulin concentration for each treatment test compared to the reference test (*p* < 0.05). Abbreviations: RG, regular glucose solution; NecP, nectarine dry extract powder; TP, dried tomato peel powder; EOL, ethanol extract from olive leaves.

As regards the postprandial insulin response curves (Figure 2), TB NecP and TB TP produced lower peak plasma concentrations at 30 min respect to the reference glucose solution (*p* = 0.03 and *P* = 0.04, respectively), whereas TB NecP demonstrated the lowest effect compared to TB TP (*p* = 0.03). Conversely, TB EOL led to a higher peak insulin glucose concentration compared to the reference glucose solution (*p* = 0.02).

**Figure 3:**
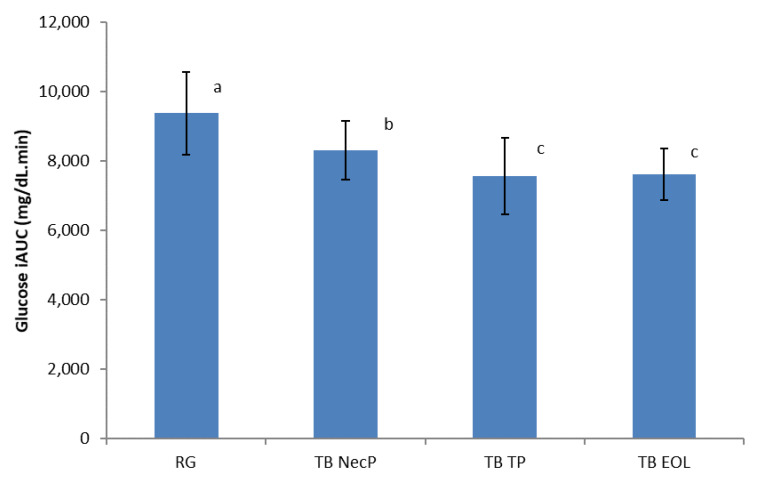
Incremental area under the curve (iAUC) postprandial glucose responses for the three treatment beverages. TB NecP (2 g of NecP, equivalent to 14 µg ABA), TB EOL (400 mg EOL, equivalent to 60.0 mg oleuropein), and TB TP (2 g TP, equivalent to 1.0 mg total carotenoids) compared to the reference glucose solution. Data are mean ± SD. ^abc^ Mean values with different superscript letters are significantly different by the Tukey–Kramer multiple comparison test (*p* < 0.05). Abbreviations: RG, regular glucose solution; NecP, nectarine dry extract powder; TP, dried tomato peel powder; EOL, ethanol extract from olive leaves.

The total glucose response over 150 min was expressed as the postprandial glucose incremental area under the curve (iAUC) ignoring the area under the baseline using the trapezoidal rule [35,36]. All of the three TB produced lower postprandial glucose iAUC compared to the reference glucose solution (TB NecP versus RG, 8317 mg/dL.min versus 9378 mg/dL.min, *p* = 0.02; TB TP versus RG, 7558 mg/dL.min vs. 9378 mg/dL.min, *p* = 0.02; TB EOL vs. RG, 7611 mg/dL.min vs. 9378 mg/dL.min, *p* = 0.02) (Figure 3). Particularly, TB TP and TB EOL demonstrated higher effect respect to TB NecP (*p* = 0.02 and *p* = 0.02, respectively), showing no significant difference between each other (*p* = 0.48).

**Figure 4:**
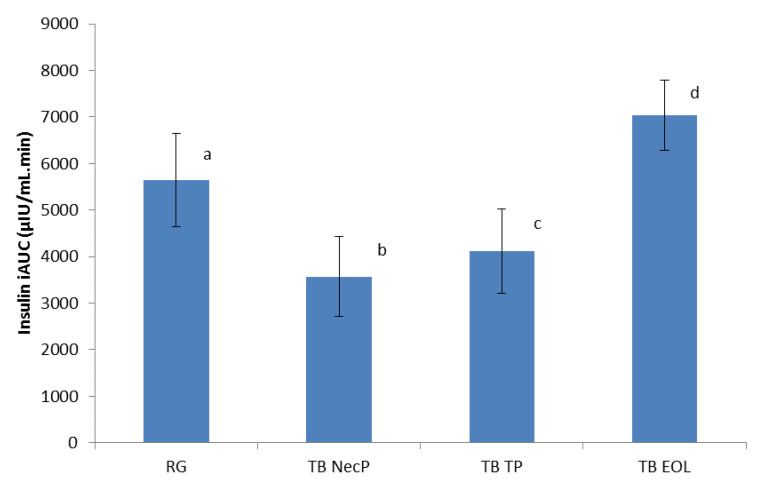
Incremental area under the curve (iAUC) postprandial insulin responses for the three treatment beverages. TB NecP (2 g of NecP, equivalent to 14 µg ABA), TB EOL (400 mg EOL, equivalent to 60.0 mg oleuropein), and TB TP (2 g TP, equivalent to 1.0 mg total carotenoids) compared to the reference glucose solution. Data are mean ± SD. ^abcd^ Mean values with different superscript letters are significantly different by the Tukey–Kramer multiple comparison test (*p* < 0.05). Abbreviations: RG, regular glucose solution; NecP, nectarine dry extract powder; TP, dried tomato peel powder; EOL, ethanol extract from olive leaves.

The postprandial insulin iAUC was calculated in the same manner as for the postprandial glucose iAUC, using the trapezoidal rule [37]. As shown in Figure 4, TB NecP and TB TP produced lower effects respect to the reference glucose solution (TB NecP versus RG, 3572 µIU/mL.min versus 5649 µIU/mL.min, *p* = 0.03; TB TP versus RG, 4116 µIU/mL.min versus 5649 µIU/mL.min, *p* = 0.02), whereas TB NecP demonstrated the lowest effect compared to TB TP (*p* = 0.03). Conversely, TB EOL led to a higher postprandial insulin iAUC compared to the reference glucose solution (*p* = 0.02).

### 3.4. Glycaemic Index and Insulinemic Index of Treatment Beverages

For the calculation of the glycaemic index (GI), the absolute iAUC glucose value for each TB was expressed as a percentage of the mean iAUC glucose values of the standard glucose solution, and the resulting values were averaged to obtain the GI value for each TB [37]. All of the three TBs led to significant reductions in GI compared to the reference glucose solution (TB NecP, *p* = 0.02; TB TP, *p* = 0.02; TB EOL, *p* = 0.02) (Table 4). Specifically, TB TP and TB EOL demonstrated higher effects respect to TB NecP (*p* = 0.02 and *p* = 0.02, respectively), showing no significant difference among each other (*p* = 0.48).

The insulinemic index (II) was calculated in the same manner as the GI, using the absolute iAUC insulin values [37]. As shown in Table 4, TB NecP and TB TP produced lower values in respect to the reference glucose solution (*p* = 0.03 and *p* = 0.04, respectively), whereas TB NecP demonstrated the lowest effect compared to TB TP (p = 0.03). Conversely, TB EOL led to a higher II compared to the reference glucose solution (*p* = 0.02).

### 3.5. Insulin Sensitivity of Subjects in Response to Reference Glucose Solution and Treatment Beverages

Insulin sensitivity of subjects was evaluated in reference to data of glucose tolerance (Figure 1) and insulin secretion (Figure 2) and expressed as values of Matsuda Indexes [38] as follows: RG, 5.99; TB NecP, 8.31; TB TP, 7.61; TB EOL, 5.98. The samples TB NecP and TB TP revealed to improve insulin sensitivity in the treatment subjects compared to the reference glucose solution.

### 3.6. Study Strength and Limitations

The major strengths of the clinical trial herein presented reside in the originality of the study and in the evaluation of the treatment effects in real-world settings. The positive results, herein reported, can inform physicians about a novel treatment/intervention which can represent a valuable support/alternative in the clinical practice. Conversely, the main limitations of our study included a small sample size of healthy participants with normal glucose tolerance and insulin sensitivity; the short-term assessment for the treatment of a chronic condition which only allowed the investigation of acute postprandial effects of the nutraceutical formulations; the lack of a dose assessment in order to define the range of minimum effective–maximum non-toxic concentrations of therapeutic interest.

## 4. Discussion

Our results revealed that all of the three nutraceutical formulations were able to significantly lower simulated postprandial glycaemia in healthy adults when added to the reference glucose solution (average reduction of glucose peak at 30 min: NecP, −9%; EOL, −17%; TP, −20%) (Figure 1). As regards the prediabetes management, previous authors reported that the occurrence of T2DM may be significantly decreased by favouring a minimum lowering of 10 GI units through diet and/or the use of food supplements [39]. The present work indicated that the acute consumption of NecP, TP, and EOL may lead to an average decrease of 10, 17, and 20 GI units, respectively (Table 3). Interestingly, our results indicated lowering effects by the treatment beverages on simulated postprandial insulinaemia, in comparison with the reference glucose solution with the exception of TB EOL (average variation of insulin peak at 30 min: NecP, −28%; TP, −36%; EOL, +20%; average variation of the II: NecP, −37 units; TP, −28 units; EOL, +20 units) (Figure 2; Table 3). Thus, the present study would highlight that two out of three of the nutraceutical formulations (mainly, NecP and TP) would be able to influence postprandial glycaemia through an insulin-saving mechanism, while EOL would preferentially modulate plasma glucose levels by stimulating insulin release. Recent scientific literature has identified ABA, carotenoids, and oleuropein, occurring in NecP, TP, and EOL, respectively, as among the natural bioactive components with the major effectiveness on animal glycemia and insulinemia [20,21,22,28,29]. Nevertheless, it must be pointed out that these nutraceutical formulations are characterised by very heterogeneous phytocomplexes. Thus, our results regarding their influence on human glycemia and insulinemia could be ascribed to a plethora of different phytochemicals rather than to an individual constituent or a specific class of compounds. In the light of these data and concerning the many different mechanisms of action of NecP, TP, and EOL, it may be hypothesised the formulation of a multi-component synergistic product, potentially useful to modulate glycaemia and insulinaemia in multifactorial patients such as diabetic subjects.

Overall, an aspect of major interest of the present study is represented by the possibility of obtaining useful nutraceutical formulations from agricultural by-products. The management of solid waste originating from agricultural processing is a serious emerging problem both in Western and developing countries. Particularly, the costs of drying, storage and shipment of by-products for their disposal are economically crucial factors. Therefore, agro-food waste is often employed as fertilizer or feed. Many attempts have been made by researchers and industries in order to face agro-food by-products. Specifically, many by-products which were first underexploited and disregarded are being increasingly converted into valuable ingredients. Some of these ingredients are commercialized and widely used by the industries as food products or as nutraceutical ingredients in functional foods [40]. Thus, our study is in line with the current worldwide trend to recover such agro-food wastes for environmental, economic, and healthy purposes.

## 5. Conclusions

Our results indicated that all of the nutraceutical formulations obtained from agro-food by-products were clinically able to significantly reduce simulated postprandial glucose levels. As regards the postprandial insulin responses, NecP and TP produced lower peak plasma concentrations, while EOL led to a higher peak insulin concentration, in respect to the reference glucose solution. Thus, the present study highlighted that two of the three nutraceutical formulations were able to influence postprandial glycaemia through an insulin-saving mechanism, while EOL would preferentially modulate plasma glucose levels by stimulating insulin release. Overall, the difference in the mechanisms of action, attributable to the main bioactive constituents of the three nutraceutical formulations, may lead to the hypothesis of a multi-component synergistic preparation which may be regarded as an innovative and promising nutritional intervention for the management of postprandial glucose homeostasis in pre-diabetic and, possibly, diabetic subjects. Undoubtedly, the present work represents a preliminary study for the evaluation of the effects of specific nutraceuticals on human glycaemia and insulinaemia which must be supported by further in vitro, in vivo, and clinical investigations to confirm the proposed results.

## Figures and Tables

**Table 1:** Content of abscisic acid in fruit thinning waste products.

	Peaches	Nectarines	Apples	Plums	Pears
µg/g FW	0.9 ± 0.5 ^a^	4.5 ± 1.5 ^c^	0.8 ± 0.3 ^a^	0.4 ± 0.2 ^b^	0.3 ± 0.1 ^b^
µg/g DW	9.5 ± 1.6 ^a^	15.0 ± 3.0 ^c^	8.1 ± 1.1 ^a^	6.5 ± 0.9 ^b^	5.5 ± 0.8 ^b^

Values are the means ± SD (*n* = 5; *p* < 0.01). ^abc^ Mean values in rows with different superscript letters are significantly different by the Tukey–Kramer multiple comparison test. Abbreviations: FW, fresh weight; DW, dry weight.

**Table 2:** HPLD-DAD determination of the main bioactive components in the nutraceutical products.

	Abscisic Acid (from NecP)	Carotenoids (from TP)	Oleuropein (from EOL)
mg/g	0.007 ± 0.004	0.5 ± 0.1	150.0 ± 5.6

Values are the mean ± SD (*n* = 5; *p* < 0.01); Abbreviations: NecP, nectarine dry extract powder; TP, dried tomato peel powder; EOL, ethanol extract from olive leaves.

**Table 3:** Baseline characteristics of randomised subjects.

Characteristics	Value
Demographics	
Age (years)	45.1 ± 15.8
Male sex (No (%))	9 (45.0%)
White ethnicity (No (%))	20 (100%)
**Clinical parameters**	
BMI (Kg/m^2^)	23.3 ± 3.4
TC (mg/dL)	190.1 ± 11.2
LDL-C (mg/dL)	98.0 ± 10.1
HDL-C (mg/dL)	57.2 ± 8.5
Triglycerides (mg/dL)	147.3 ± 12.7
Glucose (mg/dL)	82.5 ± 14.2

Values are means ± SD (*n* = 5).

**Table 4:** Glycaemic index (GI) and insulinemic index (II) for the three treatment beverages. TB NecP (2 g of NecP, equivalent to 14 µg ABA), TB EOL (400 mg EOL, equivalent to 60.0 mg oleuropein), TB TP (2 g TP, equivalent to 1.0 mg total carotenoids) in relation to the reference glucose solution (RG).

Test Solution	GI Value	II Value
RG	100 ^a^	100 ^a^
NecP	90 ± 5 ^b^	63 ± 4 ^b^
EOL	80 ± 4 ^c^	120 ± 7 ^c^
TP	83 ± 5 ^c^	72 ± 6 ^d^

Data are mean ± SD. ^abcd^ Mean values in columns with different superscript letters are significantly different by the Tukey–Kramer multiple comparison test (*p* < 0.05). Abbreviations: NecP, nectarine dry extract powder; TP, dried tomato peel powder; EOL, ethanol extract from olive leaves.

## Supplementary Materials

The following are available online at https://www.mdpi.com/2072-6643/12/5/1292/s1, supplementary file: materials and methods.
