# Supplementary material for: A Pilot Screening of Agro-Food Waste Products as Sources of Nutraceutical Formulations to Improve Simulated Postprandial Glycaemia and Insulinaemia in Healthy Subjects
Source: Nutrients. 2020 May 1;12(5):1292. doi: 10.3390/nu12051292 (PMC7282253; doi:10.3390/nu12051292)
Supplement: Supplementary file 1 [file nutrients-12-01292-s001.pdf]

Supplementary materials

Materials and methods

Sample collection and sample preparation for HPLC analyses

FTWPs (apples, pears, plums, peaches and nectarines) were collected in June 2018 at the orchards of “Giaccio Frutta” society (Vitulazio, Caserta, Italy, 41°10′N - 14°13′E), at 20-25 days after full bloom, coinciding with fruit thinning stage. Samples were immersed in liquid nitrogen (N_2), and maintained at -80 °C until analysis. Then, samples were weighed and ground in liquid N_2, and 0.5 g of each homogenized sample was suspended in 15 mL of 80% aqueous methanol containing 1% formic acid for 16 h at 4 °C in darkness under magnetic stirring. The extract was diluted to 25 mL with water, and the pH was adjusted to 2.5 with 1 M aqueous HCl, after which the samples were filtered and eluted. The eluates were then added first to SPE DSC-MCAX cartridges that had been previously washed with 2 mL of 100% methanol and equilibrated with 2 mL of water. Next, the eluates (6 mL) were added to SPE LCNH2 cartridges, also previously washed with 2 mL of 100% methanol, and equilibrated with 2 mL of water. The acidic compounds were bound to the last column, eluted with 1 mL of 5% phosphoric acid in methanol and stored at -20 °C until analysis.

Tomato peels (cultivar San Marzano) were provided in September 2018 by La Torrente S.r.l. (Angri, Salerno, Italy, 40°43′N - 14°33′E). Samples were immersed in liquid nitrogen (N_2), and maintained at -80 °C until analysis. Then, samples were weighed and ground in liquid N_2. Carotenoids were extracted in darkness, as previously described [1], with slight modifications. Approximately 1 g tomato peel powder was weighed into an 11 mL glass vial and extracted with 5 mL MeOH, briefly mixed on a vortex mixer, probe sonicated (Branson Fisher Scientific 150E Sonic Dismembrator) for 8 s, and centrifuged for 5 min at 2000 × g. The supernatant was decanted and the pellet was re-extracted with 5 mL hexanes–acetone (1:1), briefly mixed on a vortex mixer, probe sonicated for 8 s, and centrifuged for 5 min at 2000 × g. The supernatant was added to the methanolic extract, and the extraction was repeated two more times or until the pellet was colorless. To induce phase separation, 10 mL water was added to the combined supernatants, 1 mL aliquots organic layer was filtered with a 0.22 μm nylon filter (CellTreat, Shirley, MA), and stored at -20 °C until analysis.

Olive leaves (cultivar Ravece) were provided in September 2018 by Agriturismo Petrilli (Flumeri, Avellino, Italy, 41°4′N - 15°9′E). Samples were immersed in liquid nitrogen (N_2), and maintained at -80 °C until analysis. Then, samples were weighed and ground in liquid N_2. Polyphenols were extracted in darkness, as previously described [2], with slight modifications. An aliquot (1.0 g) of olive leaves powder was extracted with 20 mL ethanol (70%), stirred well by piping for homogenization for one minute, then placed in the ultrasonic bath at (75 ± 2) °C, for 30 min, filtered with 0.45 μm filters and kept at -20 °C until use. The extraction was repeated for 3 times.

HPLC-DAD analyses of samples

The chromatographic apparatus consisted of a Jasco Extrema LC-4000 system (Jasco Inc., Easton, MD) provided with the following modular components: a vacuum degassing unit, a quaternary pump, an autoinjector, a column oven, a diode array detector photodiode array detector (DAD).

As regards ABA determination, the chromatographic analyses were performed according to previous authors with slight modification [3]. The column selected was a Kinetex® C18 column 100A (250 mm x 4.6 mm i.d., 5 μm) (Phenomenex, Torrance, CA). The mobile phase was (A) acetonitrile

and (B) aqueous phosphoric acid solution at pH 3.2. The column was equilibrated, and the column temperature was maintained at 40±0.1 °C. Separation was carried out by gradient elution with a constant flow rate of 0.5 mL/min. The gradient program was as follows: 5-70% A (0−75 min). After the gradient separation, the column was re-equilibrated with 70-100% A (75−86 min) for 5 min and 100-5% A in 20 min. An injection volume of 20 µL was used for each analysis. The standard solution of ABA was prepared in methanol and chromatographed to determine the retention time for each. ABA was monitored at 265 nm.

Carotenoids were analyzed as previously described [4], with slight modifications. Separation was performed on a 4.6 × 250 mm, 3 µm particle size, C30 column (YMC Inc., Wilmington, NC) maintained at 35 °C. A total of 10 µL of purified samples was injected. A gradient using solvent A (60% MeOH, 35% MtBE, 3% water, and 2% (w/v) aqueous ammonium acetate) and B (78% MtBE, 20% MeOH, and 2% (w/v) aqueous ammonium acetate), at a flow of 1.3 mL/min, was used as follows: 100% A to 64.4% A over 9 min, 64.4% A to 0% A over 5.5 min, a hold at 0% A for an additional 3.5 min, and a switch to 100% A for the remaining 3.5 min to recondition the column. Quantification was achieved using a six-point external calibration curve of lycopene and β-carotene. Adjusted slopes were calculated for other carotenoids based on ratios of their molar extinction coefficient to lycopene, as done previously [5].

Determination of oleuropein in olive leave extracts was carried out as previously described [32], with minor modifications. A Kinetex® C18 column 100A (250 mm x 4.6 mm i.d., 5 µm) (Phenomenex, Torrance, CA) was used with the following gradient mobile phase program: A, 2.5% acetic acid; B, acetonitrile; flow rate, 1.0 mL/min,; temperature, 25 °C. chromatograms were monitored at 254 nm, for oleuropein determination. The injection volume was 20 µL.

For all of the three determination (ABA, carotenoids, oleuropein), the retention times were determined from three different injections. Peak identifications were based on retention times and standard additions to the samples; compounds were quantified according to a calibration curve that was constructed from the measurement data of the matrix-matched calibration standards.

Study population and protocol

Study participants were recruited by the Samnium Medical Cooperative (Benevento, Italy). Subjects were enrolled in January 2019. All subjects underwent a standardised physical examination, assessment of medical history (for up to five years before enrolment), laboratory examination, measurement of blood pressure and heart rate, and evaluation of BMI. Body mass index (BMI) was calculated from body height and body weight. Body fat percentage was measured using a body composition analyzer (TBF-310, Tanita Corp., Tokyo, Japan) and systolic blood pressure, diastolic blood pressure, and heart rate, were measured using a HBP-9020 (OMRON COLIN Corp., Tokyo, Japan). At each clinic visit, subjects had to complete three self-administered questionnaires on quality of life aspects, and their diaries were checked for data completeness and quality of documentation to ensure subject comprehension of the diary items. Subjects aged 18-70 years were eligible for enrolment if they had a BMI between 18–25 kg/m^2 and normal glucose tolerance. Normal glucose tolerance was assessed using the results from an oral glucose tolerance test conducted within the previous 1 month prior to participation in this study (fasting glucose <5.5 mmol/L and 2 h postprandial glucose <7.8 mmol/L). Exclusion criteria were: smoking, diabetes, serious hepatic disease (cirrhosis, hepatitis), serious renal disorders (serum creatinine >2.8 mg/dL), heart disease, family history of chronic diseases, drug therapy or supplement intake for hyperglycaemia, drug therapy or supplement intake containing ABA or carotenoids or olive polyphenols, heavy physical

exercise (>10 h/week), pregnant women, women suspected of being pregnant, women who hoped to become pregnant, breastfeeding, birch pollen allergy, and donation of blood less than 3 months before the study.

The subjects received oral and written information concerning the study before they gave their written consent. Protocol, letter of intent of volunteers, and synoptic document about the study were submitted to the Scientific Ethics Committee of AO Rummo Hospital (Benevento, Italy). The study was approved by the committee (protocol n. 28 of 15/05/2017), and carried out in accordance with the Helsinki declaration of 1964 (as revised in 2000). The subjects were asked to make records in an intake-checking table for the intervention study and side effects in daily reports. The study was a randomised, single center, randomized, double-blind trial conducted at the Samnium Medical Cooperative (Benevento, Italy).

Participants completed 6 test sessions, each on a different day, with consecutive sessions, separated by at least 1 day. Each participant tested the oral glucose solution on sessions 1, 3, and 5 and one of the three treatment beverages during each of the remaining sessions, in a random, counterbalanced order. Participants consumed the reference glucose solution on three separate occasions and each test beverage on one occasion only. Subjects were informed not to drink alcohol or perform hard physical activity 48 h prior to blood sampling. Participants maintained their usual dietary and lifestyle patterns throughout the study.

Study treatment

The reference glucose solutions and the treatment beverages all contained 75 g glucose. The three treatment beverages (TB) were prepared mixing glucose solutions with the following samples: 2 g of *NecP* (14 μg ABA) → TB *NecP*; 2 g TP (1.0 mg total carotenoids) → TB *TP*; 400 mg *EOL* (60.0 mg oleuropein) → TB *EOL*. Both TB and reference glucose solutions were served into dark jars, in order to blind subjects and researchers of the study to the different colours of the solutions mixed with the nutraceutical products. The nutraceutical products required for each treatment beverage was added into the glucose solution immediately before to being served to the subjects.

Study procedures

Participants arrived at the research centre in the morning after 12 h of fasting. All blood samples were taken in the morning and immediately after measurement of heart rate and blood pressure. Blood samples were collected from each participant before administration of the reference glucose solutions and the treatment beverages, in 3-mL EDTA-coated tubes (Becton–Dickinson, Plymouth, UK). Plasma was immediately isolated by centrifugation (20 min, 2.200 g, 4 °C). Additional finger-prick blood samples were collected at 30, 60, 90, 120 and 150 min after starting consumption of the reference glucose solutions and the TB. All seven test sessions for each participant were analysed within the same assay. All samples were stored at -80 °C until analysis. Plasma glucose levels were determined using commercially available kits from Diacron International (Grosseto, Italy). Analyses were performed on a Diacron International Free Carpe Diem. The assay sensitivity for the individual analytical determination was determined as follows: glucose, 4 mg/dL. Intra- and inter-day variations were 1.1 and 1.7% for glucose. Plasma insulin concentrations were measured using an enzyme linked immunosorbent (ELISA) assay commercial kit (InterMedical srl, Italy). The reference glucose solutions and the treatment beverages were consumed within 5 min.

Statistics

Methodology

Unless otherwise stated, all of the experimental results were expressed as mean ± standard deviation (SD) of at least five replications. Statistical analysis of data was performed by the Student's t test or two-way ANOVA followed by the Tukey-Kramer multiple comparison test to evaluate significant differences between a pair of means. The statistic heterogeneity was assessed by using Cochran's test ($p<0.1$). The I2 statistic was also calculated, and I2>50% was considered as significant heterogeneity across studies. A random-effects model was used if significant heterogeneity was shown among the trials. Otherwise, results were obtained from a fixed-effects model. SD values were calculated from standard errors, 95% CIs, p-values, or t if they were not available directly. Previously defined subgroup analyses were performed to examine the possible sources of heterogeneity within these studies and included health status, study design, type of intervention, duration, total nutraceutical dose, and Jadad score. Treatment effects were analysed using PROC MIXED with treatment and period as fixed factors, subject as random factor and baseline measurements as covariates, and defined as weighted mean difference and 95% CIs calculated for net changes in fecal and serum parameters, and blood pressure values. Data that could not meet the criteria of variance homogeneity (Levenes test) and normal distribution (determined by residual plot examination and Shapiro–Wilks test) even after log transformation were analysed by a nonparametric test (Friedman). The level of significance (α-value) was 95% in all cases ($P < 0.05$).

Analysis set

The full analysis set population included all randomised subjects, and subjects who did not fail to satisfy a major entry criterion. The per protocol set consisted of all subjects who did not substantially deviate from the protocol. This group included subjects for whom no major protocol violations were detected (for example, poor compliance, errors in treatment assignment).

Determination of sample size

In order to determine whether this study provided sufficient power to detect statistically significant differences using this design, a post hoc power analysis was performed the primary endpoints measured for all treatments (1-way repeated measures ANOVA, observed $F = 0.6596$, $\alpha = 0.05$, 1 group, $n = 20$, 5 measurements, observed correlation among measurements = 0.7977, Geisser-Greenhouse sphericity $\varepsilon = 0.4991$). Based on these values, the statistical power was 100%, indicating that the sample size was sufficient to detect statistically significant differences if they were indeed present. This resulted in $n = 10$ subjects, which was increased to $n = 20$.

Statistics

All of the experimental data were expressed as mean ± standard deviation (SD) of at least five replications. Statistical analysis of data was carried out by the Student's t test or two-way ANOVA followed by the Tukey-Kramer multiple comparison test to evaluate significant differences between a pair of means. The level of significance (α-value) was 95% in all cases ($P < 0.05$). The degree of linear relationship between two variables was measured using the Pearson product moment correlation coefficient (R). Correlation coefficients (R) were calculated using Microsoft Office Excel.

References

1. Kopec, R.E.; Cooperstone, J.L.; Schweiggert, R.M.; Young, G.S.; Harrison, E.H.; Francis, D.M.; Clinton, S.K.; Schwartz, S.J. Avocado consumption enhances human postprandial provitamin A absorption and conversion from a novel high-beta-carotene tomato sauce and from carrots. *J Nutr* **2014**, *144*, 1158-1166, doi:10.3945/jn.113.187674.

2. AlShaal, S., Karabet, F., and Daghestani, M. . Determination of the antioxidant properties of the Syrian olive leaves extracts and isolation oleuropein by HPLC techniques. *Analytical and Bioanalytical Chemistry Research* **2019**, *6*, 97-110.

3. Bosco, R.; Caser, M.; Vanara, F.; Scariot, V. Development of a rapid LC-DAD/FLD method for the simultaneous determination of auxins and abscisic acid in plant extracts. *J Agric Food Chem* **2013**, *61*, 10940-10947, doi:10.1021/jf4034305.
4. Cooperstone, J.L.; Ralston, R.A.; Riedl, K.M.; Haufe, T.C.; Schweiggert, R.M.; King, S.A.; Timmers, C.D.; Francis, D.M.; Lesinski, G.B.; Clinton, S.K., et al. Enhanced bioavailability of lycopene when consumed as cis-isomers from tangerine compared to red tomato juice, a randomized, cross-over clinical trial. *Mol Nutr Food Res* **2015**, *59*, 658-669, doi:10.1002/mnfr.201400658.
5. Cooperstone, J.L.; Francis, D.M.; Schwartz, S.J. Thermal processing differentially affects lycopene and other carotenoids in cis-lycopene containing, tangerine tomatoes. *Food Chem* **2016**, *210*, 466-472, doi:10.1016/j.foodchem.2016.04.078.
